# Cognitive understanding is a functional coordination pattern, just like swimming

**DOI:** 10.3389/fpsyg.2025.1573946

**Published:** 2025-10-22

**Authors:** Lisette De Jonge-Hoekstra

**Affiliations:** Developmental Psychology, Faculty of Behavioral and Social Sciences, University of Groningen, Groningen, Netherlands

**Keywords:** children, complex systems, coordination dynamics, affordances, cognitive development

## Introduction

Many researchers, including myself, have been intrigued by how children develop cognitive understanding. Using tasks involving, for instance, balance scales (e.g., De Jonge-Hoekstra et al., 2020; Pine et al., 2004), floating and sinking (e.g., Kloos et al., 2010; Meindertsma et al., 2014), mathematical equivalence (e.g., Alibali et al., 2007; Goldin-Meadow et al., 2009), or air pressure (e.g., Van der Steen et al., 2019), researchers have investigated how children talk, move, write, or draw when they go from less to more advanced explanations for how a task works. Researchers' ideas about cognitive understanding are predominantly based on information processing models (e.g., Case, 1985; Gordon and Ramani, 2021; Rescorla, 2025; also see next Section). In this Opinion paper, I will provide an alternative account of cognitive understanding, based on a complex systems, coordination dynamics, and affordances perspective.

## Theoretical backgrounds (a short version)

Information processing models refer to any model whereby information comes into the system (i.e., input), is processed somehow in the system, and—based on this processing—the system generates output (e.g., American Psychological Association, 2018; Gordon and Ramani, 2021; Rescorla, 2025). One way this processing can be done is in relation to cognitive understanding. To give an example of this, a child is asked to predict whether a balance scale will tilt or stay even, with weights of the same mass at a different distance from the middle (input). The child's cognitive understanding is that the balance scale will tilt toward the side with the heaviest weight. Combining the equal weights in the balance scale problem with this cognitive understanding (processing), the child predicts that the balance scale will stay even (output). From an information processing perspective, cognitive understanding is thus something abstract, within a child (often “within their head”) that is more or less stable. However, from a complex systems, coordination dynamics and affordances perspective, cognitive understanding is something different (which I will argue in the next section). I will very briefly introduce these three (related) perspectives; a more elaborate description can be found in (De Jonge-Hoekstra 2021).

Complex systems are systems that consist of multiple components, typically at multiple scales of a system, which interact and spontaneously coordinate over time by means of self-organization (e.g., Kelso, 1995; Smith and Thelen, 2003; Thelen and Smith, 2007; Van Geert, 1998, 2009; Van Orden et al., 2003). The systems' interacting components self-organize into global patterns, which can also change over time. Coordination dynamics refers to the coupling between multiple components, that leads them to adjust their actions to each other. Often these coupled systems and their components behave as if they were one—a synergy (e.g., Haken, 1977; Kelso, 2013; Latash, 2008; Strogatz, 2003; Turvey, 2007; also see Warren, 2006). A synergy is a functional grouping of systems that “work together” and self-organize in the service of a particular “goal” (Kelso, 2013; Latash, 2008; Turvey, 2007). Furthermore, systems are coupled to their environment. Affordances are possibilities for action which the environment offers to the system, thereby matching its capabilities (e.g., Adolph and Kretch, 2015; Gibson and Pick, 2000; Gibson, 1966, 1979). Children need to learn to perceive and realize affordances (Adolph and Kretch, 2015; Gibson and Pick, 2000).

Building on the complex systems, coordination dynamics and affordances perspectives, a recent review (Adolph and Hoch, 2019; also see Adolph, 2020; Adolph et al., 2018; Newen et al., 2018) summarizes the characteristics of *motor* development as embodied, embedded, enculturated and enabling. Embodied refers to the fact that the current specifics of the body determine possibilities for action. Embedded implies that the environment opens up and constrains possibilities for action. Enculturated indicates that motor development is shaped by social and cultural forces. Lastly, enabling means that each new skill opens up a whole new range of opportunities to learn other skills, and thereby can bring about a developmental cascade. Following previous researchers (e.g., Kloos and Van Orden, 2009; Thelen and Smith, 1994, 2007), I am convinced that there is no clear or absolute distinction between motor and cognitive development. This means that these characteristics of motor development thus also apply to *cognitive* development in general, and cognitive understanding in specific.

For *pragmatic* reasons, I will loosely define motor development and cognitive development in line with how they are commonly differentiated within research (also see e.g., Rietveld and Kiverstein, 2014). I refer to motor development as the development of adaptive behavior that involves directly observable movements, such as rolling, sitting, crawling, walking, and grasping. I refer to cognitive development as the development of adaptive behavior which is less directly observable—and is therefore often thought to rely on stable, internal representations—such as language, reasoning, imagining, and problem solving. The development of these adaptive behaviors, regardless of whether they are more or less directly observable, can be viewed as skill learning.

Skill learning can be seen as a change at the level of the interaction between a child and their environment, and is related to constraints (Baggs et al., 2020). (Baggs et al. 2020) describe the example of learning to walk. When a child initially learns to walk, the hands of her parents or a baby walker constrain her posture, so that she is able to walk (with support). Furthermore, constraints are provided by a stable surface to walk on, and a (preferably) good view of her surroundings. When the child becomes a more experienced, more efficient, and better walker, the constraints that enable her to walk change: While the stable surface and good view remain important, she has learned how to appropriately constrain her posture by herself, without having to rely on external support. This example shows how skill learning involves a coalescence of organismic (e.g., postural stability), environmental (e.g., solid surface), and task (e.g., parents' hands) constraints, in line with Newell (e.g., 1985). Importantly, the distinction between task and environmental constraints is heuristic in nature, and thus not absolute, but fluid (e.g., Thelen and Smith, 1994; Rietveld and Kiverstein, 2014). Any skill is constituted by constraints at—equally important—different levels, that cannot be separated without changing the skill and its function. Furthermore, skillful behavior is often regarded as optimally navigating many constraints at different levels (e.g., Rietveld and Kiverstein, 2014; Thelen, 2000). This is true for both motor skills and—as I will argue—cognitive understanding.

## Cognitive understanding is a functional coordination pattern

Based on the framework above, I propose that cognitive understanding within cognitive development is the equivalent of what a motor skill is within motor development. This entails that—similar to motor skills—cognitive understanding is a functional coordination pattern. Functional hereby means that it arises when a particular child (organismic constraints) is in a physical and social environment with particular characteristics (task/environmental constraints), such as when an adult asks them to explain about a particular task (e.g., De Jonge-Hoekstra et al., 2020; Meindertsma et al., 2014; Pine et al., 2004; Van der Steen et al., 2019). Depending on the specifics of the environment, cognitive understanding can take many forms, such as talking and gesturing, but also writing on paper, or hands-on problem solving. Similarly, also motor skills come in many different forms, such as walking, running, climbing, or swimming, depending on the environment that someone is in and the particular motor problem one is confronted with, such as moving on a horizontal surface, slanting, vertical surface, or in the water, respectively. This suggests that any form of cognitive understanding, just like any particular form of motor skills, only exists for a specific child doing a concrete task in a specific environment.

Opponents of such a view typically emphasize that viewing cognitive understanding about a particular concept as being similar to a motor skill ignores that cognitive understanding, at least in part, is abstract, symbolic, disembodied and ungrounded (for an overview, see Halford and Andrews, 2011). This expresses that cognitive understanding about a particular task, once it is well-developed, is supposed to happen “in someone's head”, and is thereby relatively independent from the specific environment that someone is in or in which the understanding emerged. However, I would like to challenge the idea that a motor skill is any less (or more) abstract or “in someone's head” than cognitive understanding about a particular task, using the example of swimming.

**Figure 1:**
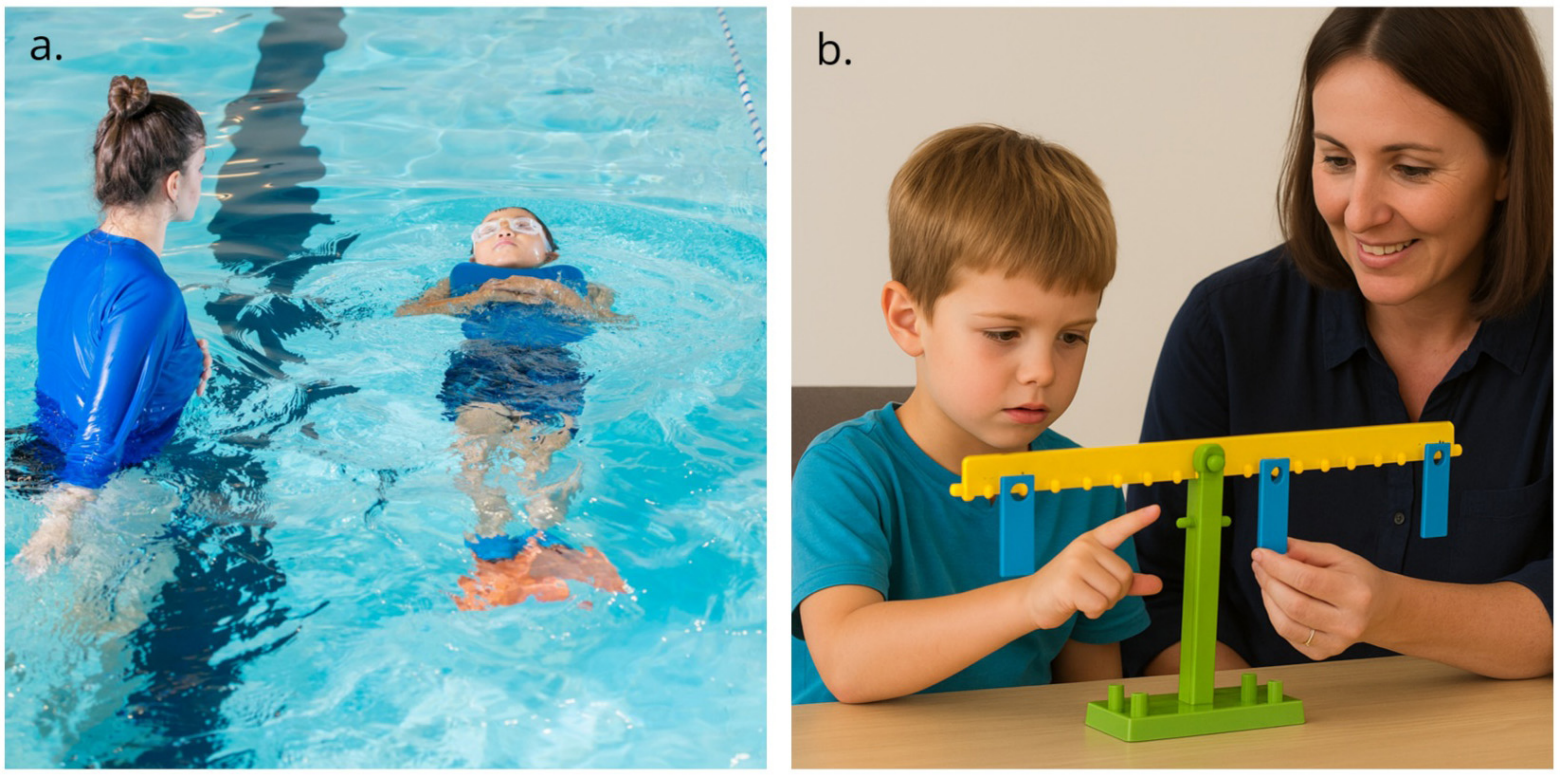
Two functional coordination patterns: **(a)** Swimming (in a pool) and **(b)** Cognitive understanding (about a balance scale). **(a)** was made by Jacob Yavin from Pexels: https://www.pexels.com/photo/swimming-school-12918910/. **(b)** was created using ChatGPT 5.0, https://chatgpt.com/, and subsequently adapted. The prompts for ChatGPT 5.0 can be found as supplementary material: https://osf.io/7mfud.

Few people would disagree that swimming is a motor skill that depends just as much on the specifics of the environment (task/environmental constraints) as that it depends on a person's capability to adjust to that in a very typical way (organismic constraints). This specific environment is a pool of water large enough for a person to move about in. Swimming on land is, strictly speaking, impossible, because the resistance of air is much lower than the resistance of water, while a floor, on the other hand, is much too resistant. Furthermore, flapping your arms and legs in the air in a pattern that looks like swimming will not get you anywhere and is thus not functional (unless your aim was to make other people laugh). Swimming thus only exists and can be concretely defined in the water. In addition, learning to swim usually starts with the help of floating attributes and under guidance of a swimming teacher (see Figure 1a). Throughout learning to swim, children increasingly learn to coordinate many components of their body so that they stay afloat and move forwards or backwards while being in the water. When you have learned to swim, we expect you to be able to swim whenever you are in the water. However, when you are not in the water and are thus not swimming, we do not think that you are not a skilled swimmer anymore. We typically do not ask “where your skill of swimming went”. No one considers it to be abstract or in your head, when you are not in the water.

Similar to swimming, cognitive understanding about a particular task only exists and can be concretely defined when a child is in a particular physical and social environment. For example, talking and gesturing about balance scale problems only happens when a child (organismic constraints) is in a situation in which a balance scale and weights are present and an adult asks them to explain about balance scale problems (task/environmental constraints) (see Figure 1b). If a child would do a similar coordination pattern while playing hide and seek, this would give away their location, and would thus not be functional. Furthermore, having learned to correctly (from the perspective of the adult) explain about balance scale problems entails paying attention to, speaking, and gesturing about both mass of the weights and distance from the fulcrum whenever a child is in a situation that requires them to do so. This is thus similar to a skilled swimmer being able to swim whenever they are in the water. I therefore assert that asking “where the cognitive understanding about balance scale problems went” when a child is not in that particular situation is just as meaningful, or rather meaningless, as asking “where the skill of swimming went”.

One last counterargument, which is in favor of cognitive understanding being fundamentally different from motor skills, is that cognitive understanding about a particular task transfers to many other situations (e.g., Goldin-Meadow, 2015), while this is not the case for motor skills. However, this argument disregards that the ability to adaptively use a motor skill in an increasing number of diverse situations is inherent to learning a motor skill (e.g., Adolph, 2020; Adolph et al., 2018; Adolph and Hoch, 2019). With regard to the previous example of swimming, while children typically learn to swim using floating attributes, and in calm waters—such as a swimming pool—later on they will learn to swim in water with waves, or currents, such as in a sea or river. On the other hand, adverse circumstances, such as heavy clothing or stormy waters, will make swimming impossible for even the most skilled swimmers.

Moreover, cognitive understanding is known to be grounded and highly sensitive to environmental circumstances. I will illustrate this with the famous example of the A-not-B error. The A-not-B error pertains to a classical Piagetian task, in which a toy is repeatedly hidden at a location A (the A-trials), where the child subsequently and correctly finds the toy. After a number of A-trials, the toy is hidden at location B. Children between 7 to 12 months old have been found to continue searching at location A, instead of location B. This has been coined as the A-not-B error (Piaget and Cook, 1954). Piaget attributed the error to the idea that children at that age have not yet developed the concept of object permanence. However, a series of studies, inspired by complex dynamical systems theory, showed that particular circumstances make the A-not-B error disappear in 10 month old children, while other circumstances elicit the A-not-B error in older children (Smith et al., 1999; Spencer et al., 2001; Thelen et al., 2001; Schöner and Thelen, 2006; Schöner and Dineva, 2007). To be specific, a salient visual difference between the locations, as well as a change in posture (i.e., sitting vs. standing) made younger children correctly search at location B during B-trials (Smith et al., 1999), for example. Furthermore, a longer waiting time between hiding the toy at location B and searching for the toy elicited the A-not-B error in children who were older than 12 months (Spencer et al., 2001). This example of the A-not-B error again shows that the theoretical perspectives of complex systems, coordination dynamics, and affordances are equally useful for capturing cognitive development and motor development.

## Discussion

Following the above argument, researchers should treat cognitive understanding as a functional coordination pattern that emerges and self-organizes from the interaction between the child and their environment (also see Kloos and Van Orden, 2009; about cognition being soft-assembled), instead of something abstract and “in the head”. Given the continuity between cognitive and motor development, researchers should investigate how characteristics of both the environment and children (i.e., constraints at different levels) make opportunities for cognitive understanding appear or disappear in a wide range of tasks (as was done for the A-not-B error; Smith et al., 1999; Spencer et al., 2001; Thelen et al., 2001; Schöner and Thelen, 2006; Schöner and Dineva, 2007). To gain further insight into how such opportunities appear and disappear, these studies should combine qualitative (i.e., descriptive) and quantitative (i.e., quantification of instability) methods to investigate the dynamics of the (transitions between) different functional coordination patterns that constitute cognitive understanding (in line with e.g., Anastas et al., 2011; Castillo et al., 2017; De Jonge-Hoekstra et al., 2021; Stephen et al., 2009; Wallot et al., 2019). Only by integrating the complex systems, coordination dynamics and affordances perspectives will we be able to move toward truly understanding cognitive understanding, which promises to have important consequences for how we teach children.
